# The Proteome of Extracellular Vesicles Released from Pulmonary Microvascular Endothelium Reveals Impact of Oxygen Conditions on Biotrauma

**DOI:** 10.3390/ijms25042415

**Published:** 2024-02-19

**Authors:** Wolfgang Schaubmayr, Beatrix Hochreiter, Eva Hunyadi-Gulyas, Louise Riegler, Katy Schmidt, Akos Tiboldi, Bernhard Moser, Klaus U. Klein, Katharina Krenn, Gisela Scharbert, Thomas Mohr, Johannes A. Schmid, Andreas Spittler, Verena Tretter

**Affiliations:** 1 Department of Anesthesia, General Intensive Care and Pain Medicine, Medical University of Vienna, 1090 Vienna, Austriabeatrix.hochreiter@meduniwien.ac.at (B.H.); katharina.krenn@meduniwien.ac.at (K.K.);; 2 Laboratory of Proteomics Research, HUN-REN Biological Research Centre, 6726 Szeged, Hungary; gulyas.eva@brc.hu; 3 Department of Orthopedics and Trauma Surgery, Medical University of Vienna, 1090 Vienna, Austria; 4 Core Facility of Cell Imaging and Ultrastructure Research, University of Vienna, 1090 Vienna, Austria; 5 Department of Thoracic Surgery, Medical University of Vienna, 1090 Vienna, Austria; 6 Institute of Cancer Research, Department of Medicine I, Comprehensive Cancer Center, Medical University of Vienna, 1090 Vienna, Austria; 7 Institute of Vascular Biology and Thrombosis Research, Medical University of Vienna, 1090 Vienna, Austria; johannes.schmid@meduniwien.ac.at; 8 Department of Surgery and Core Facility Flow Cytometry, Medical University of Vienna, 1090 Vienna, Austria; andreas.spittler@meduniwien.ac.at

**Keywords:** pulmonary endothelium, oxygen, extracellular vesicles, tissue factor, angiotensin-converting enzyme, vesicle proteomics

## Abstract

The lung can experience different oxygen concentrations, low as in hypoxia, high as under supplemental oxygen therapy, or oscillating during intermittent hypoxia as in obstructive sleep apnea or intermittent hypoxia/hyperoxia due to cyclic atelectasis in the ventilated patient. This study aimed to characterize the oxygen-condition-specific protein composition of extracellular vesicles (EVs) released from human pulmonary microvascular endothelial cells in vitro to decipher their potential role in biotrauma using quantitative proteomics with bioinformatic evaluation, transmission electron microscopy, flow cytometry, and non-activated thromboelastometry (NATEM). The release of vesicles enriched in markers CD9/CD63/CD81 was enhanced under intermittent hypoxia, strong hyperoxia and intermittent hypoxia/hyperoxia. Particles with exposed phosphatidylserine were increased under intermittent hypoxia. A small portion of vesicles were tissue factor-positive, which was enhanced under intermittent hypoxia and intermittent hypoxia/hyperoxia. EVs from treatment with intermittent hypoxia induced a significant reduction of Clotting Time in NATEM analysis compared to EVs isolated after normoxic exposure, while after intermittent hypoxia/hyperoxia, tissue factor in EVs seems to be inactive. Gene set enrichment analysis of differentially expressed genes revealed that EVs from individual oxygen conditions potentially induce different biological processes such as an inflammatory response under strong hyperoxia and intermittent hypoxia/hyperoxia and enhancement of tumor invasiveness under intermittent hypoxia.

## 1. Introduction

Mechanical ventilation with supplemental oxygen is frequently necessary to avoid hypoxemia in critically ill patients. Despite being life-saving, this treatment has the potential to add trauma to the lungs due to the build-up of pressure forces and alveolar overdistention (barotrauma, volutrauma), the cyclic collapse and reopening of different lung areas (atelectrauma) and the release of mediators, that can induce inflammation or other detrimental injury to the lungs and other organs via circulation (leading to biotrauma) [1]. Biotrauma is a major cause of multiple organ dysfunction (MODS) in this context. The ventilatory forces and the applied oxygen are independent triggers of potentially harmful effects, as high concentrations of oxygen per se cause oxidative stress and disturbance of the redox balance. In addition, atelectasis induces cyclic collapse and the reopening of lung areas, thereby initiating oxygen oscillations with high amplitudes. These oxygen oscillations are propagated via circulation to distant organs [2,3] and have been hypothesized to bear the potential for harm [4]. Oscillations in the hypoxic range are observed in the context of other diseases, such as obstructive sleep apnea syndrome (OSAS), asthma, and some tumors and have been shown to induce pathological developments [5].

Mediators of biotrauma are primarily recognized as soluble factors, such as cytokines and other pro-inflammatory proteins, but cells under stress also release extracellular vesicles (EVs), that might mediate detrimental effects, but alternatively, protective effects via their cargo (proteins, lipids, nucleic acids). Research into the characterization and the biological effects of EVs has rocketed in the last few years. Initially observed in the 1960s as “platelet dust” [6], it took the research community several decades to recognize and to learn to understand their role in intercellular communication. The general term “extracellular vesicles” has been determined in 2011 to comprise all entities with a lipid bilayer found in the extracellular space. EVs have been shown to transfer information between cells in the form of mRNA, miRNA and proteins, thereby maintaining physiological functions or spreading and ameliorating disease states. For instance, EVs play an important role in the immune response and in the intercellular communication of cells in the nervous system [7]. Also, tumor cell-derived EVs have been proven to deliver the information of drug resistance to endothelial cells [8] or enhance metastasis [9]. The efficiency of this communication is a new hope for the development of novel drug delivery systems, which might have superior qualities to other vehicles like liposomes [10]. As EVs occur in body fluids, such as saliva, milk, blood and urine, they can easily be obtained from liquid biopsies and be analyzed for biomarkers [11]. Especially challenging has been their heterogeneity with regard to origin, release mechanism, cargo, target cells and uptake mechanisms [12]. Depending on their size, mode of biogenesis, and sedimentation properties, EVs formerly have been classified into exosomes (<200 nm, endocytic origin, <100,000 g sedimentation), microparticles (200–1000 nm, shed from the plasma membrane; 10,000–20,000 g sedimentation) and apoptotic bodies (50 nm–5 mm, shed from dying cells, 2000–10,000 g sedimentation) [13]. However, the characterization of subpopulations has proven to be rather difficult, as overlaps of markers in different vesicle populations have been detected. Therefore, the International Society for Extracellular Vesicles (ISEV) regularly issues guidelines to support a standardized methodology for the isolation and analysis of these vesicles [14].

Established methods to characterize the vesicle cargo are flow cytometry and omics technologies, such as (quantitative) proteomics and next-generation sequencing for nucleic acids [13,15,16,17].

Recent studies have revealed that pathological cyclic stretch in acute respiratory distress syndrome (ARDS) activates leukocytes and induces the release of leukocyte-derived EVs with a pro-inflammatory cargo, that has the potential to induce remote organ endothelial injury. Also, patients with hyperinflammatory ARDS have been shown to exhibit elevated levels of neutrophil- and endothelium-derived EVs [18].

In our study we pursued the hypothesis that altered oxygen conditions including oxygen oscillations could function as potential contributors to biotrauma and we investigated the role of extracellular vesicles released by lung microvascular endothelial cells in an in vitro model. We aimed to characterize these vesicles in response to the direct oxygen pattern impact in the absence of other injurious mechanisms due to mechanical and physical factors.

## 2. Results

### 2.1. Proteomic Analysis of Extracellular Vesicles

To isolate EVs from supernatants and to reduce the accumulation of soluble proteins at the same time, we used 100 kDa cut-off ultrafiltration [19] after 48 h of exposure to different oxygen (O_2_) conditions. The experience of our and other groups has shown that 48 h is an optimal duration for the secretion of sufficient EVs for analysis without affecting stability. Ultrafiltrates with 30 μg protein content were analyzed by quantitative LC-MSMS.

**Figure 1:**
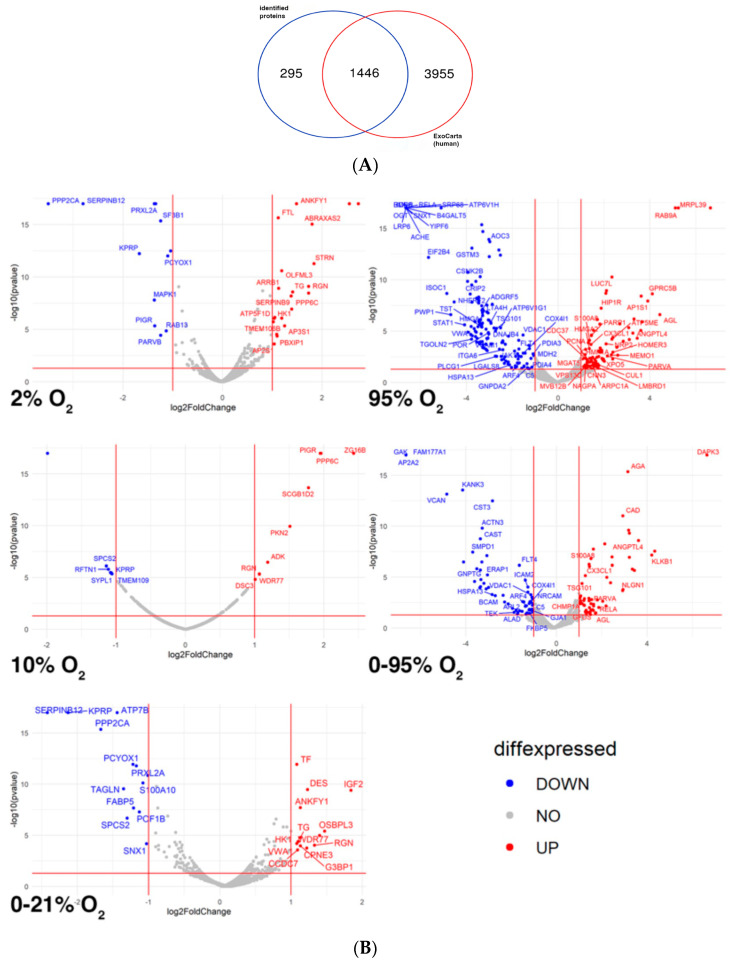
(Semi-)quantitative proteomic analysis of extracellular vesicles released from HMVEC-L in response to different hypoxic and hyperoxic O_2_ conditions. (**A**) Venn diagram comparing identified proteins to human exosome-associated proteins listed in ExoCarta database. (**B**) Volcano plots of differentially expressed proteins with significantly down-regulated (in blue color) or up-regulated (in red color) abundance.

Using this method, about 1750 proteins could be identified in the ultrafiltrate. Among those, all the accepted marker proteins, which are known to be enriched in all EV fractions, respectively, such as CD9, CD63, CD81, flotillin, heat-shock proteins, Alix, Tsg101, annexins were detected, as well as marker proteins indicating the endothelial origin of the vesicles, such as CD31, CD54, CD105, CD144, CD146, von Willebrand factor (vWF) and others. For a full results list see Supplemental Data S1 (Tab “all proteins”). When comparing all identified proteins to human exosome-associated proteins listed in the ExoCharta database (www.exocarta.org; accessed on 7 February 2024), we found an overlap of 1446 proteins (see Venn diagram in Figure 1A).

To investigate O_2_-condition-specific changes in vesicle cargo, a quantitative assessment of protein levels was performed. Volcano plots in Figure 1B reveal that proteins were significantly up- or downregulated under the different hypoxic and hyperoxic O_2_ conditions relative to normoxia (21% O_2_). For a full list of significantly changed proteins under all O_2_ conditions compared to normoxia see Supplemental Data S1.

### 2.2. Functional Enrichment Analysis

Proteins found to be significantly changed in abundance (differentially expressed proteins, DEPs) in the EV fractions of different O_2_ exposures were uploaded including their “fold up/downregulation” onto the web-based platform NetworkAnalyst 3.0 (www.networkanalyst.ca) and compared with the protein-interactome of the STRING database to calculate zero- and first-order networks with the DEPs serving as seeds. The resulting networks were imported into Cytoscape software for further analyses and visualization. The STRING app was applied to compute functional enrichments indicating altered signaling pathways (Supplemental Data S2a and S2b)

When considering all of the detected proteins in the ultrafiltrate, the assigned protein–protein interaction network analysis identified EV cargo impacting the immune system, hemostasis, platelet activation, signaling and aggregation, cell cycle, apoptosis, developmental biology, cytokine signaling, membrane trafficking, and mRNA processing. The most prominently affected pathways include NOTCH, Toll-like receptors, PDGF, MAPK, FGFR, TGFß and SCF/KIT. Enrichment analyses of DEPs using the GO and Reactome databases are summarized in Supplemental Data S2b.

**Figure 2:**
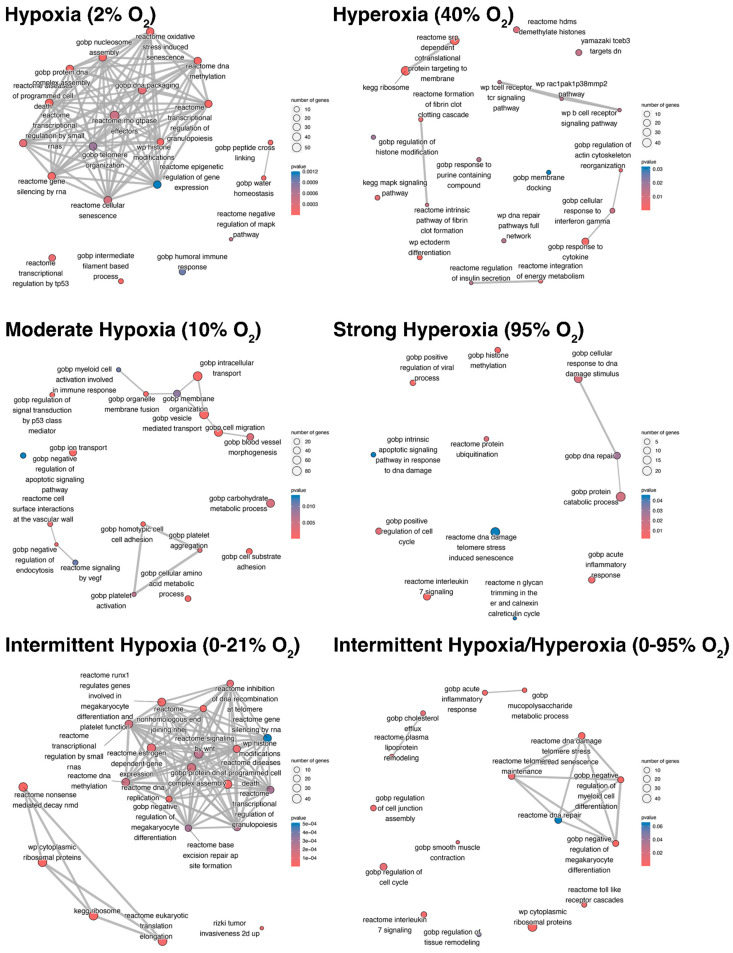
Enrichment Maps of GSEA.

Further analysis by Gene Set Enrichment Analysis (GSEA) revealed majorly enriched processes as depicted in Figure 2. The full results list of GSEA is found in Supplemental Data S3.

DEPs from proteomic analysis of EVs obtained after exposure of HMVEC-L to different O_2_ conditions were used as input for GSEA. Nodes represent enrichment terms according to the respective database (GO, KEGG, Reactome et al.), and edges indicate overlapping gene sets. Nodes are color coded according to their obtained *p*-value as shown in the individual legend. The plots show the most representative enriched terms.

### 2.3. Release of Different Types of Vesicles as Detected by Flow Cytometry

**Figure 3:**
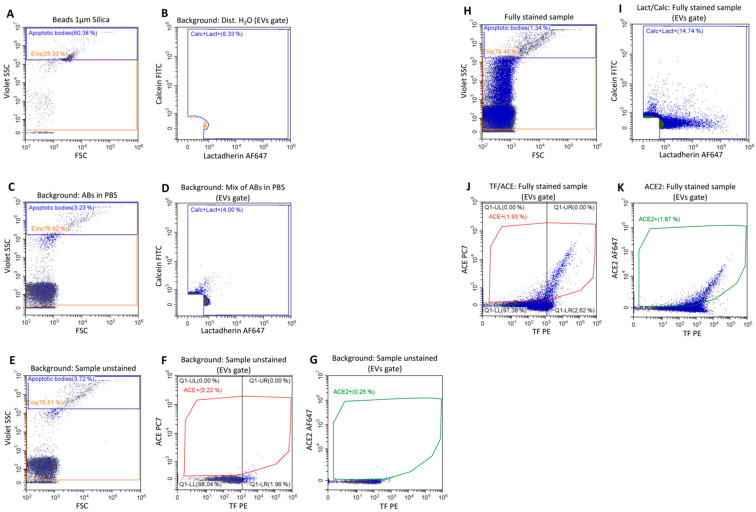
Gating strategies for the quantification of extracellular vesicles stained for calcein/lactadherin/TF/ACE/ACE2. (**A**) Silica beads (1 µm) were used to standardize the Violet SSC (VSSC) gain settings and exclude large EVs (=remaining apoptotic bodies and /or EV aggregates) in Violet SSC vs. FSC scattering. Signals below the EVs gate show the electronic noise of the cytometer. Events in the gate “Apoptotic bodies” were excluded from the analysis of the data. (**B**) The supernatant of the centrifuged reagents (antibody, lactadherin, and calcein-AM) was used at the same concentration diluted in PBS as used in the sample staining. (**C**) To check for background noise, the reagent mixture was triggered for calcein (FITC) and lactadherin (AF647) and measured for 2 min, resulting in a maximum background of 120 events/sec. Subsequently, the main visible background population was gated and defined as Calc+Lact+ population. (**D**) Shows the “rounded” gate with background signals from distilled water. (**E**) Unstained samples were measured in the Violet SSC vs. FSC scatter and EVs’ gated background signals were transferred to (**F**). The Q1-LR region was set to define the negative TF control population and the ACE+ gate was set to define the negative ACE control expression. (**G**) Gate ACE2+ designated the negative ACE2 control population. (**H**) Fully stained samples were measured in Violet SSC vs. FSC scatter and gated using the EVs’ gate. (**I**) Lactadherin and calcein positive events were gated as described above and (**J**) TF positive signals in Q1-LR as well as ACE positive events in ACE+ were measured. (**K**) Similarly, ACE2-positive EVs were measured as gated in (**H**,**I**).

EVs of an endosomal or plasma membrane origin (exosomes, MVs) were detected by two different flow cytometry protocols. Exosome-like EVs of endosomal origin are frequently enriched with the marker proteins CD9, CD63 and/or CD81. However, not all exosomes express equal amounts of all three markers. Expression levels have been shown to depend on the cell (sub)type and might also vary between donors [20]. Also, some members of the tetraspanin-family proteins are located on the cell surface and might also be present in larger plasma membrane-derived (micro)vesicles. Therefore, it is not possible to achieve a strict separation into exosomes and microvesicles with these marker proteins. We used an antibody cocktail of CD9, CD63 and CD81 in conjunction with the lipid membrane dye vFRed^TM^ to detect and quantify a vesicle population that contains mostly exosomes. We also analyzed another population of vesicles that are phosphatidylserine-positive and therefore plasma membrane-derived by their capability to bind lactadherin in conjunction with the vesicle dye calcein. The design of the gating strategy used is depicted in Figure 3 and explained in more detail in the Section 4.

**Figure 4:**
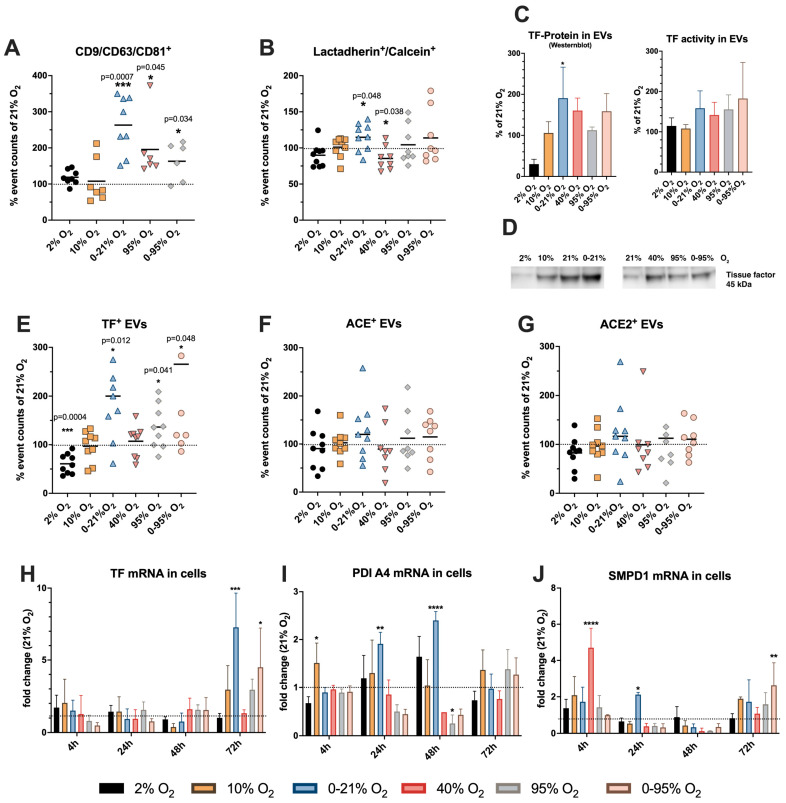
Quantitative analysis of extracellular vesicles by flow cytometry. Two different flow cytometry protocols were applied as described in the main text to detect (**A**) exosome-like vesicles and (**B**) phosphatidylserine-positive (lactadherin-binding) microvesicles in ultrafiltrate after 48 h exposure to different O_2_ conditions. Output variables (positive events/μL) of each O_2_ condition were related (positive events/μL) under normoxia (21% O_2_). The diagrams depict the mean (black line) and individual values of each donor. The 100% value (dotted line) represents no change compared to 21% O_2_ and this was used as a reference value for one-sample *t*-test. (**C**) Western blot analysis of tissue factor (TF) protein in vesicle preparations and TF activity test (Zymuphen MP-TF Kit). (**D**) Representative Western blot of TF antigen in extracellular vesicles. (**E**) Tissue factor (**F**) angiotensin-converting enzyme (ACE) (**G**) ACE2-positive events in EV ultrafiltrate (% relative to 21% O_2_). mRNA expression of (**H**) tissue factor, (**I**) protein disulfide-isomerase (PDI) A4, (**J**) acid sphingomyelinase (SMPD) 1 in HMVEC-L after exposure to different O_2_ conditions over time (RT-qPCR). The dotted line indicates no change relative to normoxia (21% O_2_). Two-way ANOVA (Dunnett’s multiple comparisons test) * *p* < 0.05; ** *p* < 0.01; *** *p* < 0.001; **** *p* < 0.0001.

Mean ± SD values of exosome-like vesicles positive for the markers CD9/CD63 and/or CD81 in % relative to 21% O_2_ were as follows: 118.6 ± 19.9 (2% O_2_); 107.9 ± 60.9 (10% O_2_); 263.0 ± 79.4 (0–21% O_2_); 195.6 ± 88.0 (95% O_2_); and 163 ± 53.1 (0–95% O_2_) (Figure 4A).

Mean ± SD values of (plasma membrane-derived) phosphatidylserine-positive EVs in % relative to 21% O_2_ were as follows: 89.9 ± 16.3 (2% O_2_); 101.0 ± 14.0 (10% O_2_); 114.8 ± 19.1 (0–21% O_2_); 85.5 ± 16.1 (40% O_2_); 104.4 ± 26.6 (95% O_2_); and 113.8 ± 37.1 (0–95% O_2_) (Figure 4B).

Nanoparticle Tracking Analysis (NTA) also gave an estimation of size distribution of extracellular vesicles in the individual samples. All O_2_ treatments revealed a dominant peak at approximately 160–180 nm with a prevalence of 96–99% of vesicles, indicating the majority of vesicles being larger exosomes. A total of 0.6–2.3% of vesicles were around 30 nm in size and 0.1–0.5% were larger than 650 nm. Differences between treatment groups were not significant. Sizes measured by NTA can certainly be only estimations but were grossly supported also by our electron microscopy analyses (see below).

Previous studies have shown that EVs released from endothelial cells can also express tissue factor (TF) on their surface [21] and that this expression can be enhanced under certain conditions of stress, such as hyperoxia [22]. TF activity is tightly regulated by several different mechanisms to prevent pro-coagulative events during normal physiological conditions. In our analyses we addressed the amount of vesicle-bound TF from different perspectives: (1) amount of TF-positive vesicles released (Figure 4E); (2) amount of TF-antigen protein in the vesicles (Figure 4C,D); and (3) TF-activity in the vesicles (Figure 4C). As TF activity regulation is complex, these analyses are not necessarily directly comparable.

Determining vesicle-bound TF activity using the Zymuphen MP-TF Kit, we found an upward trend under intermittent hypoxia and under hyperoxic conditions (Figure 4C). The sensitivity and specificity of such assays have been a matter of discussion and modified custom-designed assays have been described in the literature [23]. Western blot analysis for total TF protein in the ultrafiltrates of EVs revealed an increase under intermittent hypoxia (0–21% O_2_; *p* < 0.05) and an upward trend under (intermittent) hyperoxia (40% O_2_, 95% O_2_, 0–95% O_2_) compared to normoxia (21% O_2_) (Figure 4C,D).

To estimate the abundance of TF-positive vesicles, we co-stained the vesicle preparation with lactadherin, calcein and TF antibody. Co-staining with tetraspanin markers was not feasible due to incompatible fluorescent dyes.

Mean ± SD values in % relative to 21% O_2_ of TF-positive (micro-)vesicles were 60.8 ± 20.4 (2% O_2_); 97.0 ± 31.2 (10% O_2_); 199.5 ± 92.3 (0–21% O_2_); 107 ± 31.9 (40% O_2_); 136.4 ± 44.7 (95% O_2_); 265.7 ± 213.1 (0–95% O_2_); and n = 9 (Figure 4E).

It has been shown in previous studies that EVs can contain the plasma membrane proteins ACE and ACE2, two components of the renin-angiotensin system (RAS), which play a role in diverse (patho)physiological contexts [24,25]. These proteins have not been detected in our proteomic analysis, but were detected in a smaller fraction of vesicles by flow cytometry using specific validated antibodies. Figure 4F,G show the quantification of ACE and ACE2 positive events (in % relative to 21% O_2_). In these panels, it is evident that samples from different donors were rather variable with regard to their ACE^+^ and ACE2^+^ EV response to different O_2_ conditions, especially under intermittent hypoxia and (intermittent) hyperoxia exhibiting obvious up- or downregulation, respectively.

### 2.4. Cellular Expression of mRNA of Tissue Factor and Its Activity Regulators SMPD1 and PDI

We evaluated the induction of TF expression in HMVEC-L after exposure to different O_2_ conditions. qRT-PCR revealed an O_2_-condition- and time-dependent increase in expression of TF mRNA in cell lysates (Figure 4H). Fold change mean ± SD values relative to 21% O_2_ only changed significantly under some conditions after 72 h exposure: 1.019 ± 0.301 (2% O_2_); 2.953 ± 1.654 (10% O_2_); 7.273 ± 2.381 (0–21% O_2_); 1.338 ± 0.234 (40% O_2_); 2.943 ± 0.734 (95% O_2_); and 4.500 ± 2.722 (0–95% O_2_) (n = 3–4). Protein disulfide isomerase (PDI) and acid sphingomyelinase (SMPD) are two functionally independent enzymes that regulate TF activity (see Discussion). PDI protein (subtype A4) was readily detectable and quantifiable in the proteome of EVs from all three donors; SMPD1 levels were low in EVs and could be detected only in the sample from one donor. Fold change values of protein levels were as follows (after 48 h exposure): 2% O_2_: PDIA4 0.94 ± 0.96; SMPD1 1.52; 10% O_2_: PDIA4 0.91 ± 0.98; SMPD1 1.87; 0–21% O_2_: PDIA4 1.32 ± 0.41; SMPD1 1.25; 95% O_2_: PDIA4 0.44 ± 0.03; SMPD1 0.87; 0–95% O_2_: PDIA4 0.62 ± 0.36; and SMPD1 0.077.

We determined the expression of these enzymes also in HMVEC-L cells over a longer time range: fold change values relative to 21% O_2_ are shown in Table 1.

### 2.5. Release of Different Types of Vesicles as Detected by Transmission Electron Microscopy Presence of Tissue Factor

**Figure 5:**
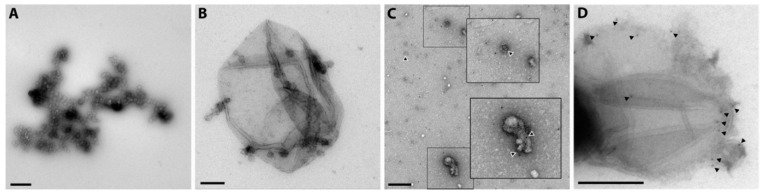
Transmission electron microscopy of negatively stained extracellular vesicles. (**A**) Small vesicles from a preparation subjected to 21% O_2_. (**B**) Image from a 10% O_2_ incubation showing one very large and several small adherent vesicles. (**C**,**D**) Immunogold labeling (5 nm) of extracellular vesicles from a 21% O_2_ exposure labeled with anti-tissue factor antibody. The inserts in panel (**C**) show clustered vesicles labeled with gold particles. Arrow heads in panels (**C**,**D**) point out the location of gold particles indicating the presence of tissue factor. Scale bars = 200 nm.

Transmission Electron Microscopy (TEM) is regarded as a standard methodology to verify the presence of vesicles and to estimate their size. Here we used a standard protocol for negative staining and identified vesicles of smaller (100 nm) and larger (250 nm) sizes, most likely representing exosome-like vesicles and plasma membrane-derived particles (Figure 5A,B). A quantitative assessment of the size distribution of vesicles from different O_2_ treatments is not feasible with this method, as differential adherence of vesicles to the formvar-coated grid is possible and cannot be accurately determined. Also, larger vesicles tend to collapse.

We also performed immunogold labeling for TEM using a TF antibody and were able to detect specific gold particles in small (Figure 5C), as well as larger (Figure 5D) vesicles indicating TF cargo on the surface of all vesicle fractions.

### 2.6. Functional Implications of Tissue Factor in Extracellular Vesicles: Non-Activated Thromboelastometry

**Figure 6:**
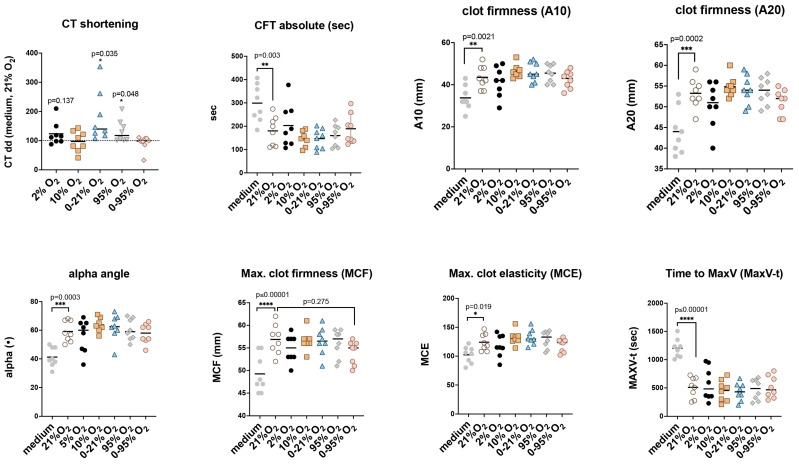
Non-activated Thromboelastometry (NATEM) analysis of the impact of extracellular vesicles on blood coagulation. (One-sample *t*-test or One-way ANOVA: * *p* < 0.05; ** *p* < 0.01; *** *p* < 0.001, **** *p* < 0.0001).

Even very small amounts of TF bound to EVs can have an impact on blood coagulation and can be monitored by thromboelastometry, especially NATEM. In NATEM assays, coagulation is activated only by the addition of calcium to reverse the effect of citrate from the blood collection tube and therefore this assay is especially sensitive to endogenous coagulation activators such as TF (factor III). Changes in hemostatic mechanisms evaluating the influencing factors from both the extrinsic and intrinsic coagulation pathways can be detected with NATEM reliably and with high sensitivity. Examples of its application in the clinical setting are the detection of risk factors for portal vein thrombosis due to endothelial damage and EV release [26], diffuse intravascular coagulation (DIC) [27], and also previous preclinical experiments assessing EV-bound TF released from endothelial cells [21]. Here we used NATEM analysis to assess the possible functional effects of altered TF content of EV preparations after different O_2_ conditions (Figure 6).

NATEM analysis was performed with fresh blood and 10μL ultrafiltrate (approx. 5 × 10^6^ vesicles as determined by nanoparticle tracking assay). Extracellular vesicles were released from HMVEC-L during 48 h exposure to different O_2_ conditions. Graphs show evaluated parameters such as shortening of Clotting Time relative to medium and 21% O_2_ (CT dd), Clot Formation Time (CFT), alpha angle, Amplitude after 10 min and 20 min beginning from CFT (A10, A20); Maximum Clot Firmness (MCF), and Time to Maximum Velocity (MaxV-t). For statistical evaluation we used a one-sample *t*-test for CT dd and One-way ANOVA and Dunnett’s multiple comparisons test for all other parameters with 21% O_2_ as the reference group. Black lines indicate mean values.

Individual NATEM analyses of EV preparations from different cell donors were performed on different days with varying blood donors. EV preparations from different O_2_ exposures from the same cell donor were analyzed in parallel at the same time with the 21% O_2_ exposure. To eliminate different assay conditions (such as blood donors), Clotting time (CT) values were normalized relative to the empty secretion medium control sample and relative to the respective 21% O_2_ sample (=CT dd in %) (Figure 6). Mean ± SD (in %) including all *p*-values were as follows: 2% O_2_: 123.5 ± 39.6 (*p* = 0.137); 10% O_2_: 97.2 ± 35.5 (*p* = 0.83); 0–21% O_2_: 179.5 ± 86.5 (*p* = 0.035); 95% O_2_: 132.6 ± 36.5 (*p* = 0.048); and 0–95% O_2_: 92.3 ± 25.3 (*p* = 0.41). All other NATEM parameters were significantly different from the empty secretion medium, but showed no difference between O_2_ conditions.

## 3. Discussion

Endothelial cells are key players in lung homeostasis acting to ensure an anti-inflammatory and anti-coagulative phenotype. Dysregulation of this homeostasis is a central issue in ARDS, a condition with still limited treatment options and high mortality [28]. Release of EVs not only from endothelial cells, but also from platelets, neutrophils, monocytes, and lymphocytes carrying bio-active cargos frequently worsen the condition and serve as biomarker candidates [29,30]. Besides other soluble factors, these vesicles are considered to contribute to biotrauma, which can ultimately result in multiple organ failure (MOF), a primary cause of death in patients with ARDS. As EVs are very heterogeneous with regard to their composition (dependent on their biogenesis, origin and triggering stimuli), their analysis is highly sophisticated and interpretation of their physiological impact is often ambiguous. In this study we tried to limit complexity by focusing on the isolated response of human pulmonary microvascular endothelial cells to different oxygen conditions, to discern the impact of oxygen from other influencing factors, such as mechanical stretch, infection, and inflammation and to eliminate additional crosstalk with other cell types. EVs are either derived from endosomes or directly from the plasma membrane and their cargo is determined by the physiological condition of the originating cell, but also selective loading mechanisms seem to exist. It is still unclear how selectively these vesicles are taken up by target cells and to what extent the protein and nucleic acid cargo actually will impact the receiving cell. In this study we combined analytical techniques with bioinformatic analyses and functional tests to study the impact of different oxygen conditions on EV composition and to deduce possible downstream effects.

Bioinformatic evaluation of proteomic analysis revealed enrichment of the following pathways of particular interest. Hypoxia: complement and coagulation pathways, platelet homeostasis, DNA packaging and methylation, immune responses, telomere organization and senescence, glycolysis and fatty acid metabolism, tight junctions, endothelial cell migration, VEGF-VEGFA2 and TNFα pathway activation and negative MAPK regulation. Intermittent hypoxia: cell cycle checkpoints, DNA damage, response to ER stress, signaling by interleukins (IL-1), cell response to calcium ions, ferroptosis, ribosome biogenesis, HIF1, NOTCH, and WNT pathways. Strong hyperoxia: DNA damage response and DNA repair, acute inflammatory response, cell cycle, catabolic processes and protein ubiquitination, IL-7 signaling, and N-glycan trimming (calnexin-calreticulin cycle). Intermittent hypoxia/hyperoxia: acute inflammatory response, Toll-like receptor and IL-7 signaling, smooth muscle contraction, cell junction assembly, negative regulation of megakaryocyte and myeloid cell differentiation, cholesterol efflux and DNA damage response/DNA repair, and telomere maintenance.

To analyze the heterogeneous vesicle preparations in more detail, we used elaborated flow cytometry settings to distinguish the exosome-like fraction (CD9+/CD63+/CD81+ enriched) from plasma membrane-derived EVs (lactadherin/calcein positive). We found a significant increase under intermittent hypoxia (0–21% O_2_) for MVs, which was even higher (2.5-fold) for exosome-like particles. The exosome-like particles were further significantly increased under strong hyperoxia (95% O_2_) and intermittent hypoxia/hyperoxia (0–95% O_2_). Intermittent hypoxia is a hallmark for obstructive sleep apnea syndrome (OSAS), a disease, where a recurrent upper airway collapse during sleep leads to periodical de- and reoxygenation. Patients develop systemic inflammation, hypertension, atherosclerosis, cardiovascular diseases and metabolic disorders. In the light of recent knowledge gained in the field of extracellular vesicle research and their role in disease, it is very likely that EVs will also play their part as mediators of remote tissue injury in OSAS [31]. It has also been shown that intermittent hypoxia induces the release of tumor-derived extracellular vesicles that enhance an immunosuppressive status in macrophages [32]. Animal experiments in the context of mechanical ventilation have proven that oxygen oscillations with different amplitudes not only have a local impact in the lungs, but are transmitted via the circulation to remote organs including the brain [2,4,33]. It can be speculated that an increased release of extracellular vesicles from various tissues in the body can have a self-propagating effect on systemic inflammation.

Activated endothelium has repeatedly been shown to release MVs carrying factor III (TF). Despite its absence in the results table of our proteomic analysis, we analyzed our concentrated vesicle fractions for this cargo by flow cytometry, and in addition for the RAS enzymes ACE and ACE2. We found positive vesicles in all samples, even in the normoxic (21% O_2_) sample, which might be due to the fact that endothelial cells display a certain degree of activation in cell culture. Electron microscopy imaging revealed TF+ vesicles of varying size implicating its presence in MVs as well as in exosome-like particles. Quantitative flow cytometry analysis showed that TF+ EVs were significantly increased under both types of oscillations (0–21% O_2_; 0–95% O_2_). Regulation of TF expression is complex and includes various upstream ligands such as cytokines, G-protein coupled receptor agonists and transcription factors, known to be activated by oxidative stress and inflammation [34,35]. Interestingly, cellular TF mRNA was only significantly increased after 72 h exposure to oscillations, while enhanced vesicle secretion was already detected at 48 h, indicating a separate mechanism of vesicle loading compared to transcriptional activation. Molecular events leading to TF loading into vesicles have been identified to some extent [36]. However, mechanistic investigations into the regulation of TF expression and TF-positive vesicle release triggered by different O_2_ conditions will require many more detailed studies and cannot be addressed here. NATEM analysis revealed that the vesicle populations under all conditions were able to shorten the CT value relative to vesicle-free medium, indicating pro-coagulative activity. Interestingly, despite the increased amounts of TF+ vesicles under 0–21% O_2_ and 0–95% O_2_, only 0–21% O_2_ showed enhanced CT shortening relative to 21% O_2_. Under physiological conditions, plasma levels of TF are low and its activity is further dampened by encryption, which is mechanistically explained by the delicate regulation of TF interaction with sphingomyelin and phosphatidylserine [36]. TF decryption has been shown to be regulated by several mechanisms, including acid sphingomyelinase (SMPD1) and protein disulfide isomerase (PDI) [37]. Our proteomic analysis detected significant amounts of PDI (subtype A4) in the vesicle preparations; however, EV levels of SMPD1 were low. Vesicle levels of PDIA4 as quantified in the proteomic analysis of EVs correlated well with CT shortening in NATEM (upregulation under 0–21% O_2_ and downregulation under 0–95% O_2_); also, SMPD1 levels detected only in one donor were significantly upregulated under 0–21% O_2_ and downregulated under 0–95% O_2_. The activation status of vesicle TF might also depend on the activation status acquired already in the originating cell and might be influenced by enrichment of vesicles with cofactor lipids, such as phosphatidylserine and phosphatidylethanolamine [38]. Therefore, we quantified expression levels of SMPD1 and PDIA4 mRNA in the HMVEC-L cells. Indeed, we found an increased PDIA4 expression until 48 h exposure to 0–21% O_2_, but not for 0–95% O_2_, and also an increased expression of SMPD1 until 24 h 0–21% O_2_, but not 0–95% O_2_. Analyses of EV lipid composition could give additional explanations for variations of TF activity under different O_2_ conditions but this was beyond the scope of this study. TF activity is further physiologically regulated by TFPI, which is tethered to the endothelium via proteoglycans. Synthesis of glycosaminoglycans is reduced by proinflammatory cytokines, thereby impairing TFPI function.

An interesting question is what clinical implications these findings could have. Circulating TF+ EVs have proven to be a good biomarker for thrombosis and disseminated intravascular coagulation (DIC) in various diseases [29]. In some types of cancers, the activity of TF+ EVs is related to the incidence of venous thromboembolism, while in other types of cancer, even a correlation between TF+ EVs and mortality has been detected. Injection of TF+ EVs into mice has been shown to cause acute thrombocytopenia and shock [39]. ARDS patients frequently reveal elevated TF+ EVs in BALF and plasma, which are an active part of the “immunothrombosis concept” of ARDS with the formation of microthrombi and a further enhancement of inflammation by signaling via protease-activated receptors (PARs) [40].

Plasma TF+ EVs are prognostic factors for mortality risk, progression to severe disease and ICU admission in patients with COVID-19 and can be used as a risk stratification tool for clinical decision-making [41,42]. SARS-CoV2 infection actively induces TF-mediated coagulation by activation of SMPD1, which could therapeutically be restricted by functional inhibitors of the enzyme [43]. In addition, it is noteworthy that thrombogenicity of EVs is not necessarily linked to TF activity and may depend on the releasing cell type and pathological status. It has been shown by addition of platelet-derived EVs to vesicle-free human plasma, that treatment with annexin V, but not with anti-TF blocking antibodies could prevent thrombin generation, indicating an important role of exposed phosphatidylserine [44]. Besides TF, exosomes from COVID-19 patients have been shown by proteomic analysis to convey pro-inflammatory cargo that was effective in triggering inflammatory responses in cells of distant organs [45]. Generally, the proteome of EVs in COVID-19 shows abnormalities, such as complement activation and platelet hyperreactivity [46], and increased loads of vWF, UPAR, and ADAMTS13 that correlates with higher levels of the thrombotic marker, D-dimer, and increased disease severity. In our study, vWF was shown to be upregulated as an EV component under 0–21% O_2_ but downregulated under 95% O_2_ and it may also have an important impact on pro-coagulative activity of EVs.

Calcium regulates EV release, as it is a necessary activator of SNAREs, but also the formation of MVs due to a reorganization of the cytoskeleton, cleavage by calpain and the activation of PKC [30,47,48]. Elevations of intracellular calcium by various mechanisms are a feature of chronic intermittent hypoxia [49] and coincidently might be related to an increased release of vesicles under O_2_ oscillations. However, increased pro-coagulative activity was only detected from EVs under intermittent hypoxia. Despite ablated pro-coagulative activity in TF+EVs from hypoxic/hyperoxic oscillations, these vesicles will prevail in circulation and one may speculate, if (encrypted) TF may be reactivated by blood-borne factors later on. Changes in the properties of EVs from endothelial cells under intermittent hypoxia have also been analyzed by Sanz-Rubio [50].

Part of the vesicles released from endothelial cells also contain components of the RAS, especially ACE and ACE2, which might play a special role regarding the development of hypertension [51]. ACE2-containing EVs have been shown to influence the infectivity of SARS-CoV2 [52]. We also assessed possible changes in the EV content of these RAS enzymes, but we could not find a clear trend in any direction, despite some strong responders among the cell donors. Especially RAS-modifying drugs that are commonly prescribed for the therapy of arterial hypertension might have had an impact on levels of ACE+ or ACE2+ EVs.

A bias in our experimental design may have been serum removal in the EV secretion medium, which is known to prevent MV release; this may be the reason why we hardly observed any change in the amount of MVs under the different O_2_ conditions [53]. However, serum starvation has been considered a better approach than using vesicle-free FBS for downstream proteomics, as serum proteins can bind non-specifically to EVs and reduce MS sensitivity [54].

In summary, we were able to show in this study that different oxygen conditions have a distinct impact on the quality and quantity of EVs released from lung microvascular endothelium and thereby might critically influence other pathological processes including biotrauma. Methodological progress in analyses of EV heterogeneity might help in the future to support decision making in finetuning of therapeutical interventions in disease states, where oxygenation is a limiting factor.

## 4. Materials and Methods

### 4.1. Cell Culture and Gas Exposure

HMVEC-L from individual donors were cultivated in EGM2-MV medium containing all supplements from LONZA’s bullet kit (#CC-3202) (LONZA, Basel, Switzerland) including 5% FBS and were expanded within 3–5 passages. Prior to exposure, medium was replaced with a secretion medium (basal medium EGM2 containing only antibiotics; serum-free). Six-well cell culture plates with 2 mL secretion medium/well were exposed to different gas conditions in a custom-designed exposure unit as described in [55]. Gas exposure was performed for 48 h at 37 °C using the following gas conditions: 21% O_2_, 2% O_2_, 10% O_2_, 40% O_2_, 95% O_2_, 0–21% O_2_, 0–95% O_2_, all with 5% CO_2_, rest N_2_.

### 4.2. Isolation of Extracellular Vesicles

After gas exposure, cell culture supernatants were harvested and centrifuged for 10 min at 200× *g* in order to remove larger debris. Apoptotic bodies were removed by centrifugation for 10 min at 2000× *g*. Finally, vesicles were concentrated by ultrafiltration using 100 kDa filters (Amicon Ultra-15; Millipore, Burlington, MA, USA) at 3200 g with 3 washes, in order to remove non-vesicle soluble proteins. A total of 12 mL supernatant was concentrated to 250 μL final volume and washed 3 times with PBS in order to minimize the presence of soluble proteins.

Vesicles were used fresh for flow cytometry and non-activated thromboelastometry (NATEM) analysis and were stored at −70 °C for proteomics analysis and at −20 °C for tissue factor activity assays.

### 4.3. Proteomic Analysis

Quantitative proteomic analysis was performed from EV ultrafiltrates of 3 donor samples exposed to 2% O_2_, 10% O_2_, 0–21% O_2_, 95% O_2_ and 0–95% O_2_ containing 30 μg protein using tandem mass tags (TMT) labeling and liquid chromatography-tandem mass spectrometry (LC-MSMS) analysis on an Orbitrap Fusion Lumos Tribrid mass spectrometer (ThermoFisher, Waltham, MA, USA). Due to limited cell material, samples from exposure to 40% O_2_ could be obtained only from 2 donors. Data analysis was also performed on results from these 2 donors but this needs to be interpreted with more caution for reasons of limited statistical evaluation. The proteomic analysis was performed after an exposure time of 48 h, which is the time needed to harvest a sufficient number of extracellular vesicles with regard to secretion and stability.

#### 4.3.1. Sample Preparation and LC-MSMS Analysis

The protein content of the isolated exosome samples was digested with trypsin using the S-Trap micro spin columns according to the vendor’s protocol (https://protifi.com/pages/protocols, accessed on 31 May 2023). Briefly: Samples were mixed with 6 μL of 20% SDS, sonicated for 5 min, dried in speed-vac concentrator and resuspended in 23 µL of 50 mM TEAB (pH: 8.5). Proteins were reduced with 5 mM TCEP @55 °C for 15 min and alkylated with 20 mM MMTS @RT for 10 min. The pH was decreased below 1 with the addition of phosphoric acid and proteins were trapped to the S-Trap™ micro spin column. After washing, 1 µg of Trypsin (Pierce Trypsin Protease, MS grade) was added and incubated at 47 °C for 2 h. Tryptic peptides were eluted and dried for before TMT labeling. The resulting peptide mixtures were labeled with TMT 10plex isobaric label reagents (Thermo Scientific, Waltham, MA, USA), 0.8 mg, product number: 90113 Lot number: WI319139) according to the standard labeling protocol:(https://www.thermofisher.com/document-connect/document-connect.html//MAN0016969_2162457_TMT10plex_UG.pdf, accessed on 31 May 2023).

Briefly, dried sample digests were dissolved in 50 µL of 100 mM TEAB buffer and 20 µL of TMT reagent (0.8 mg resuspended in 41 µL anhydrous acetonitrile) was added and incubated at room temperature for 1 h. The excess reagent was quenched by the addition of hydroxylamine. Equal amounts of each sample (corresponding to the same donor) were combined and dried down before fractionation.

High pH Reversed-Phase Peptide Fractionation Kit (PIERCE, ThermoScientific, Waltham, MA, USA) was used to prefractionate the pooled TMT labelled samples before LC-MSMS analysis according to the vendor’s protocol. Briefly, the sample was loaded to the column in 0.1% TFA and eluted successively with 5%, 10%, 12.5%, 15%, 17.5%, 20%, 22.5%, 25% and 50% acetonitrile containing 0.1% triethylamine. Eluates were dried down, redissolved in 1% TFA and loaded on Evotips (Evosep Biosystems, Odense, Denmark) according to the vendor’s protocol.

#### 4.3.2. Mass Spectrometry Analysis

Samples were analyzed by LC-MSMS using an Evosep One system (Evosep Biosystems) on-line coupled to an Orbitrap Fusion Lumos Tribrid (Thermo Scientific) mass spectrometer. The 15-samples-per-day method with its preprogrammed gradient was applied using an endurance column (EV-1106 column, 15 cm × 150 µm, 1.9 µm); the flow rate was 220 nL/min. Peptides eluted from the column were analyzed in 3 s cycles selecting the most abundant multiply charged ions (z = 2–6, *m/z* range: 400–1600) for HCD fragmentation (normalized collision energy: 38%) following each MS1 scan. Both MS and MS/MS spectra were collected in the Orbitrap analyzer with a resolution of 120,000 or 50,000, respectively.

#### 4.3.3. Data Analysis, Statistics

Database search and quantitative analysis were carried out using the Proteome Discoverer (v3.0 SP1, Thermo Scientific) software. Proteins were identified using the Sequest HT search engine with the following parameters. Database: SwissProt Homo sapiens sequences (2022.12.14. version, 20330 sequences) concatenated with a reversed version for each entry; enzyme: trypsin allowing maximum two missed cleavage sites; modifications: static: methylthio on Cys, TMT label on any N terminus and on Lysine residues; dynamic: oxidation of Met, deamidation of Gln or Asn, allowing up to 4 variable modifications/peptide; and mass accuracy: 5 ppm and 0.02 Da for precursor and fragment ions, respectively. The peptide level false discovery rate was below 1% for all samples as estimated by the incidence of reversed sequence identifications.

For quantification, the S/N (signal to noise) values of the reporter ions were used. Hyperoxic- and hypoxic-treated samples were compared to their own (same donor, same time) 21%-oxygen-treated samples in nested design after normalization to the total peptide amount per channel. Protein ratio calculations were based on pairwise ratios and background-based T-test was applied for hypothesis testing.

Statistical analysis was performed by ProteomeDiscoverer 3.0 (ThermoScientific, Waltham, MA, USA). The calculated *t*-test *p*-values and the abundance ratios were plotted on volcano plots. Significance threshold was set 0.05 for *p*-value and at least 2-fold change for protein abundance. Figures were generated using an R script, based on the instruction from https://biocorecrg.github.io/CRG_RIntroduction/volcano-plots.html, accessed on 31 May 2023.

It is important to state that samples derived from cell culture always contain proteins from the initial culture medium, especially bovine serum proteins. It is difficult to remove those by washing, even when serum-free culture medium is used as a final secretion medium, and some of them can bind unspecifically to EVs. In our proteomic analysis several bovine proteins were detected. This bias could be ameliorated by using the human proteome database for protein identification and methodologically by high pH fractionation prior to LC-MSMS analysis in order to reduce the complexity of the sample and allow deeper detection of the EV proteome and increased MS sensitivity. Some of the remaining serum proteins may still originate from the culture medium and cannot be assigned unambiguously, as there is significant homology between some human and bovine serum proteins.

### 4.4. Pathway Enrichment and Protein Network Analysis

Proteins identified in the ultrafiltrates and found to be significantly changed in relative abundances under different oxygen conditions were uploaded to the web-based platform NetworkAnalyst (3.0) [56] (www.networkanalyst.ca) (accessed on 31 May 2023) using H. sapiens (human) as species and ID (Uniprot) as identifier. These proteins represent the seeds in Zero- or First-order Networks generated using the protein interactome of the STRING database (cut-off: 700; all interactions with experimental verification). Networks were imported into Cytoscape 3.8.2. for further analyses (using the STRING app) and visualization. Functional enrichment was analyzed in the first place by using NetworkAnalyst 3.0 software and GOBP/Reactome databases.

Gene Set Enrichment Analysis (GSEA) was performed to identify enriched pathways in the proteome of EVs from different oxygen exposures. Data were imported into R [57], https://www.R-project.org, and normalized using the DEP package. GSEA was performed using the clusterProfiler package [58] with logFC as a ranking metric. Where appropriate, *p*-values were corrected according to Benjamini-Hochberg [59].

### 4.5. Flow Cytometry Analysis of EVs

Determination of EVs via flow cytometry analysis was performed using concentrated cell supernatants after 48 h gas exposure. Ultrafiltrates from 6 to 9 donors were analyzed by flow cytometry to quantify events positive for calcein (=vesicles) and lactadherin (phosphatidylserine-positive EVs; MVs), as well as CD9/CD63/CD81-positive events positive for vFRed^TM^ dye membrane stain (exosome-like vesicles). MVs were subtyped with regard to their surface-exposed TF, ACE, and ACE2 cargo.

EVs enriched in markers CD9/CD63/CD81 (exosome-like EVs) were stained and analyzed using vFC vesicle flow cytometry EV analysis assay (Cellarcus) and a CytoFLEX LX flow cytometer (Beckman Coulter, Brea, CA, USA) equipped with Violet Side Scatter detection and calibrated using vCal^TM^ nanoRainbow beads according to the manufacturer’s instruction.

#### EV Staining Protocol for Flow Cytometry

Antibodies used for staining EVs were pre-mixed and centrifuged for 10 min at 17,000× *g*. Supernatant was subsequently used for staining procedures: anti-human TF-PE (LSBio: LS-C751060), anti-human ACE-PE/Cy7 (Biolegend, San Diego, CA, USA #344208), and anti-human ACE2-AF750 (R&D Systems, Minneapolis, MN, USA; #AB9332). A total of 25 μL PBS was added to 10 μL concentrated vesicle sample and 6.5 μL antibody mixture, incubated on ice for 60 min and after that combined with 10 μL bovine lactadherin-AF647 (1:10) (HTI) and calcein. After a further 30 min incubation, samples were fixed with 50 μL 4% para-formaldehyde diluted with 850 μL PBS. All reagents were titrated prior to staining procedure to determine the optimal reagent concentration.

For the quantitation of tetraspanin-positive vesicles we used the vFC^TM^ vesicle flow cytometry EV analysis assay (Cellarcus, La Jolla, CA, USA #CBS4H-1PC7) and anti-human TS-cocktail (CD9, CD63, CD81; PE-Cy7 labeled) in combination with the vesicle dye vFRed^TM^ membrane stain. The kit is designed to detect and quantify vesicles down to the size of 50 nm.

Measurements were performed with a CytoFLEX LX (Beckman Coulter, Brea, CA, USA) flow cytometer equipped with five lasers (355 nm, 405 nm, 488 nm, 561 nm, and 638 nm). Daily start-up procedures and quality checks were carried out according to the manufacturer’s recommendations. To make the device more sensitive for size measurement and to get an impression of the size of measured EVs, the scatter signal was changed to the violet 405 nm laser. For this purpose, the first filter (450 nm) was switched to the lowest wavelength filter (405 nm) and size characterization was carried out with silica beads (1 µm, EV gate, Figure 3A).

Samples were stained with endothelial cell-specific markers (see staining protocol) as well as with lactadherin (LA) and calcein-AM. Prior to the staining procedure, a stock solution of formerly concentration-dependent titrated reagents was centrifuged (17,000× *g*, 10 min) to avoid detection of antibody aggregates. The scatter background signal for the mixture of centrifuged antibodies, lactadherin and calcein is shown in Figure 3. LA (AF647) binds to phosphatidylserine (PS) and is excited by the 638 nm laser and measured at a wavelength of 660 nm. Since a proportion of EVs are negative for PS, we used calcein-AM as an additional marker to detect EVs. Calcein-AM is taken up by EVs and converted into calcein (CA) by intracellular esterases. CA is then excited by the 488 nm laser and measured at 525 nm wavelength. Both substances served as “backbone” markers to identify the total amount of detectable EVs. Therefore, and as shown in Figure 3, a combination of two trigger signals were set, depending on which signal was detected earlier, either the CA signal or the LA signal. Furthermore, a region around the background signal of the reagent mixture was set and defined as subsequently Calc+Lact+ events. To demonstrate the gating more clearly, a sample containing distilled water is shown in Figure 3D, which shows the background noise of the cytometer.

The endothelium-specific markers used in the present study were Tissue factor, (TF, PE-labeled), angiotensin-converting enzyme (ACE, PC7-labeled) and angiotensin-converting enzyme 2 (ACE2, AF750-labeled).

Scattered EVs (Figure 3H) that were CA+ and/or LA+ were gated (Figure 3I) and included for subsequent analysis for TF (Figure 3J, gate Q1-LR), ACE (Figure 3J, gate ACE+) and ACE2 (Figure 3K, gate ACE2). As negative and background controls, the unstained samples were used (Figure 3E–G). Additionally, lysed controls and isotype controls were used (data not shown). All samples were measured for two minutes, and the results are reported as events/µL.

Measurements were performed at the lowest flow rate (10 µL/min) with an event rate/second below 2800. These parameters were tested in previous experiments to minimize swarm effects, which could be a serious problem in the detection of tiny particles which are much smaller than the height of the laser beam [60]. Swarm effects are phenomena in which several particles pass the laser beam at the same time. This makes the detection of single events impossible and therefore possibly underestimates the correct particle concentration. In addition, and to minimize carry-over effects between the samples, washing steps with distilled water were carried out between measurements.

### 4.6. Tissue Factor Activity Assay

Activity measurement of tissue factor on the surface of EVs was performed using the Zymuphen MP-TF Kit (Hyphen BioMed, Neuville-sur-Oise, France) according to the protocol provided with the kit. Briefly, vesicles containing tissue factor are bound to a microplate with an antibody that does not interfere with the biological activity. Factor VIIa and factor X are introduced and the tissue factor–factor VIIa complex activates factor X (Xa) in the presence of calcium ions. Factor Xa converts a chromogen into a yellow color that can be quantified at 405 nm on a spectrophotometer.

### 4.7. Nanoparticle Tracking Analysis (NTA)

For semi-quantitative assessment of vesicles after ultrafiltration, samples were diluted in PBS and analyzed on a ZetaView NTA instrument (Particle Metrix, Inning am Ammersee, Germany) with the concomitant software (version 8.04.02). The measurement mode was Size Distribution with 3 cycles and 11 positions per cycle under constant temperature conditions. The particle size range was set to 0.1 nm–1000 nm with a minimum particle brightness of 30, shutter set to 70 and sensitivity to 76.

### 4.8. Non-Activated Thromboelastometry Test (NATEM)

Blood from healthy volunteers was collected from a single venipuncture through a vacutainer system using a tourniquet. The coagulation tubes contained 0.105 M sodium citrate, resulting in a final citrate concentration of 3.2% in the collected whole blood. Measurements were performed in the freshly collected citrated blood to prevent bias by different test initiation times. A NATEM assay was performed using rotational thromboelastometry (ROTEM^®^; TEM International, Munich, Germany). For recalcification, 20 μL of 0.2 M CaCl_2_ was added to the measuring cup (Star-TEM reagent, ROTEM^®^; TEM International, Munich, Germany) before starting the analysis. Then, after a semi-automatic mixing step, 300 μL of citrate whole blood was added to the cup, followed by 10 μL fresh EV ultrafiltrate. The run time of the test was at least 60 min. The following NATEM variables were evaluated: Clotting time (CT, s), which indicates the time from the start of the measurement to the start of clotting; Clot formation time (CFT, s), i.e., the time after CT and until the formation of a 20 mm clot; the amplitude measured after 10 and 20 min (A10 and A20); the alpha angle (α) reflecting the kinetics of clot formation; the maximum clot firmness (MCF, mm), indicating the final strength of the clot; and the maximum clot elasticity (MCE), calculated as MCE = (100 × MCF)/(100 − MCF).

### 4.9. Transmission Electron Microscopy

Negative staining and immunogold labelling of the exosome preparations was essentially carried out as described in Thery et al. [61] with minor modifications and omission of the uranyl-oxalate step. In detail, 5 µL of an exosome preparation was adsorbed on formvar–carbon coated grids for 2 min and washed four times on drops of PBS for a minute each. For blocking, grids were incubated on 3% BSA in PBS for 30 min. The anti-hFaktor III antibodies (1:50; AF2329, R&D systems/Bio-Techne, MN, USA) were detected with gold-labelled (5 nm) anti-goat-IgGs (1:20; G-5528, Sigma-Aldrich, MO, USA). Both antibodies were incubated for 60 min in 0.3% BSA/PBS interrupted by six washing steps on PBS for 1 min. After six quick rinses with distilled water, grids were contrasted with 2% methylcellulose / 4% uranyl acetate (8:2) for 10 min on ice. Images were acquired with an FEI Tecnai20 electron microscope (FEI Eindhoven, NL) equipped with a 4K Eagle CCD camera and with the manufacturer’s software (TIA 3.1.2 build 4016). Brightness and contrast of the micrographs were adjusted with the level function (excluding the gamma values) of Adobe Photoshop Elements. Images were cropped to fit into panels where necessary.

### 4.10. Real-Time Quantitative Polymerase Chain Reaction

After different gas exposures, cells were lysed and mRNA was isolated using RNeasy mini plus kit (Qiagen, Hilden Germany). Reverse transcription was performed using qScript Supermix (Quanta Biosciences, Gaithersburg, MD, USA) and qRT-PCR was run on a RotorGene Q (Qiagen, Hilden, Germany) using SYBRgreen Fast Mix (Quanta Biosciences, Gaithersburg, MD, USA) and the following primers:

*Actb forward: (AGACGCAGGATGGCATGGG); Actb reverse (GAGACCTTCAACACCCCAGCC)*
*F3(TF) forward (GGCGCTTCAGGCACTACAA); F3(TF) reverse (TTGATTGACGGGTTTGGGTTC)*
*PDIA4 forward (GGCAGGCTGTAGACTACGAG); PDIA4 reverse (TTGGTCAACACAAGCGTGACT)*
*SMPD1 forward (CTGTCTGACTCTCGGGTTCTC); SMPD1 reverse (CTATGCGATGTAACCTGGCAG)*

### 4.11. Western Blot Analysis

SDS-PAGE for subsequent Western blot analysis using a 10% gel for detection of TF was performed according to the method of Glossmann and Neville [62,63]. Gels were semi-dry blotted onto a nitrocellulose membrane. Membranes were blocked with 5% non-fat dry milk in TBS-Tween for 1 h and incubated with goat anti-human Factor III antibody (1:2000; R&D Systems/Bio-Techne, MN, USA) overnight at 4 °C. Secondary antibody was a biotinylated horse anti-goat antibody (LONZA) with subsequent incubation with streptavidin-HRP (1:200; R&D Systems/Bio-Techne, MN, USA). Bands were visualized using a gel imaging system (Vilber, Marne-la-Vallée, France).

### 4.12. Statistical Analysis

Due to the high expenditure of work for proteomic analysis and the relatively large amounts of vesicles needed, only cells from three donors were analyzed by this methodology.

For NATEM and flow cytometry samples, we used vesicle preparations from 6 to 9 donors, depending on the cell material available after propagation in vitro. All variables were treated as normally distributed and are presented as mean  ±  standard deviation (SD). One-sample *t*-tests were calculated for all samples normalized to medium and 21% O_2_ condition. Mixed effect models with random intercepts were calculated to test for all other variables. For group analyses, we used One-way ANOVA followed by Dunnett post hoc test where indicated. Statistical analyses were performed using GraphPad Prism 9 (GraphPad Software, Inc., La Jolla, CA, USA) and Matlab (MATLAB and Statistics Toolbox R2023a, The MathWorks, Inc., Natick, MA, USA).

## Figures and Tables

**Table 1:** Changes of mRNA expression of PDIA4 and SMPD1 in HMVEC-L (RT-qPCR) over time.

	2% O_2_		10% O_2_		0–21% O_2_		40% O_2_		95% O_2_		0–95% O_2_	
	PDIA4	SMPD1	PDIA4	SMPD1	PDIA4	SMPD1	PDIA4	SMPD1	PDIA4	SMPD1	PDIA4	SMPD1
4 h	0.7 ± 0.01	1.4 ± 0.48	1.5 ± 0.42	2.1 ± 1.03	0.9 ± 0.10	1.7 ± 0.80	0.9 ± 0.08	4.7 ± 1.10	0.9 ± 0.07	1.4 ± 0.64	0.9 ± 0.12	1.0 ± 0.03
24 h	1.2 ± 0.40	0.7 ± 0.18	1.3 ± 0.69	0.5 ± 0.12	1.9 ± 0.2	2.1 ± 0.12 *	0.8 ± 0.3	0.4 ± 0.16	0.5 ± 0.14 *	0.4 ± 0.13	0.5 ± 0.10 *	0.3 ± 0.20
48 h	1.6 ± 0.42	0.9 ± 0.56	1.0 ± 0.54	0.4 ± 0.27	2.4 ± 0.18 *	0.3 ± 0.18 *	0.5 ± 0.01	0.1 ± 0.16	0.3 ± 0.16	0.1 ± 0.01 **	0.4 ± 0.12	0.4 ± 0.18
72 h	0.7 ± 0.19	0.8 ± 0.25	1.4 ± 0.42	1.9 ± 0.09 *	0.9 ± 0.30	1.7 ± 1.20	0.8 ± 0.17	1.1 ± 0.34	1.4 ± 0.41	1.6 ± 0.64	1.3 ± 0.35	2.6 ± 1.24

Values are mean ± SD of fold change relative to 21% O_2_ (one-sample *t*-test: * *p* < 0.05; ** *p* < 0.01).

## Supplementary Materials

The following supporting information can be downloaded at: https://www.mdpi.com/article/10.3390/ijms25042415/s1.
