# Supplementary material for: The Proteome of Extracellular Vesicles Released from Pulmonary Microvascular Endothelium Reveals Impact of Oxygen Conditions on Biotrauma
Source: Int J Mol Sci. 2024 Feb 19;25(4):2415. doi: 10.3390/ijms25042415 (PMC10889365; doi:10.3390/ijms25042415)
Supplement: Supplementary file 1 [file ijms-25-02415-s001.zip › Supplemental Data S4.pdf]

HMVEC-L Donors list:

NR.	ETHNIC	GENDER	AGE
**1**	caucasian	female	n.d.
**2**	caucasian	male	50y
**3**	hispanic	male	75y
**4**	caucasian	female	33y
**5**	hispanic	male	50y
**6**	caucasian	male	48y
**7**	hispanic	male	44y
**8**	caucasian	female	66y
**9**	caucasian	male	65y
