# Supplementary figures and images for: The Proteome of Extracellular Vesicles Released from Pulmonary Microvascular Endothelium Reveals Impact of Oxygen Conditions on Biotrauma
Source: Int J Mol Sci. 2024 Feb 19;25(4):2415. doi: 10.3390/ijms25042415 (PMC10889365; doi:10.3390/ijms25042415)

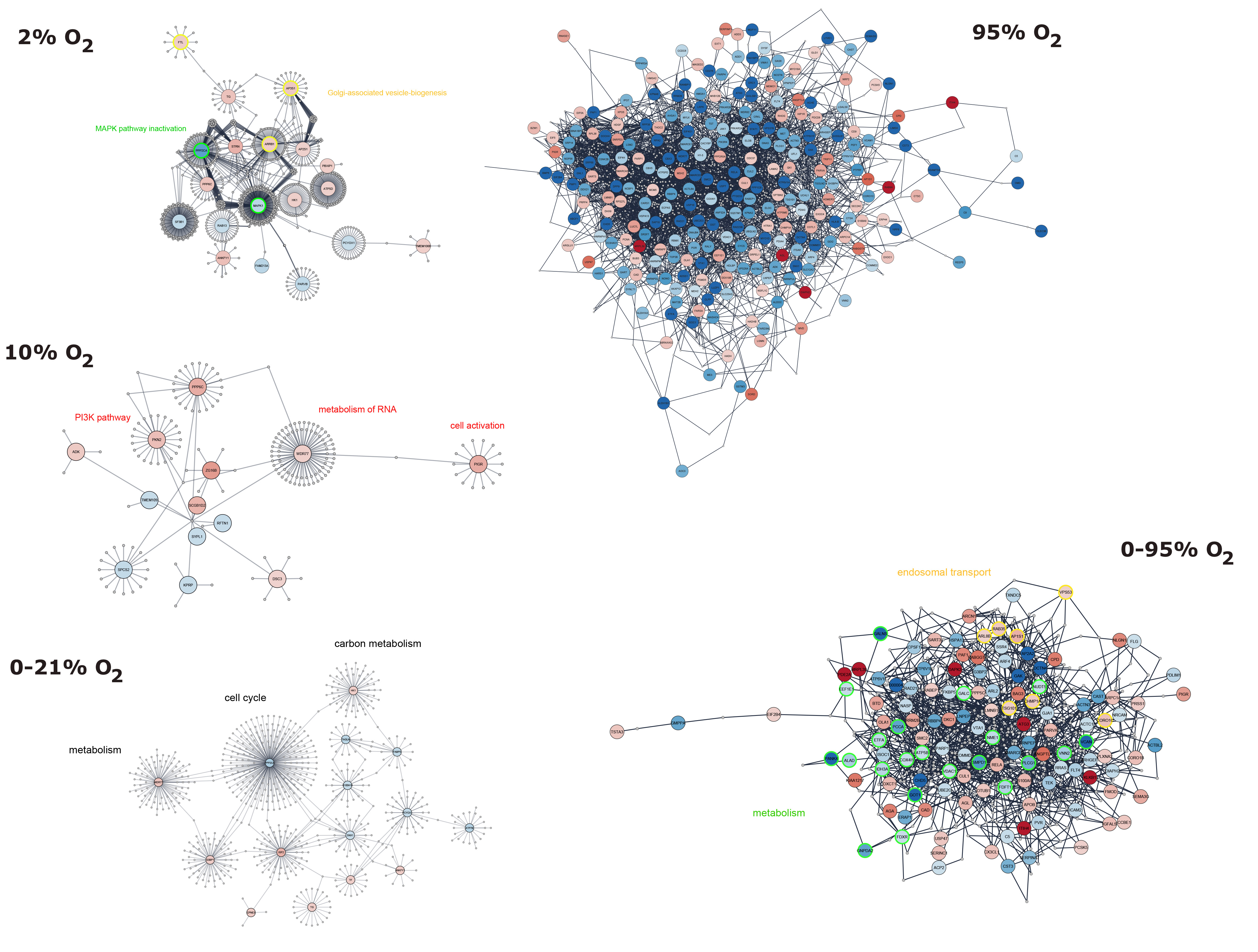

Supplement: Supplementary file 1 [file ijms-25-02415-s001.zip › Supplemetal Data S2a.jpg]
